# Overexpression of Golgi Protein CYP21-4s Improves Crop Productivity in Potato and Rice by Increasing the Abundance of Mannosidic Glycoproteins

**DOI:** 10.3389/fpls.2017.01250

**Published:** 2017-07-20

**Authors:** Hyun Ji Park, Areum Lee, Sang Sook Lee, Dong-Ju An, Ki-Beom Moon, Jun Cheul Ahn, Hyun-Soon Kim, Hye Sun Cho

**Affiliations:** ^1^Plant Systems Engineering Research Center, Korea Research Institute of Bioscience & Biotechnology Daejeon, South Korea; ^2^Department of Biosystems and Bioengineering, KRIBB School of Biotechnology, Korea University of Science and Technology Daejeon, South Korea; ^3^Department of Pharmacology, College of Medicine, Seonam University Namwon, South Korea

**Keywords:** cyclophilin, CYP21-4, golgi apparatus, glycoprotein, potato, productivity, rice

## Abstract

CYP21-4 is a novel Golgi-localized cyclophilin protein involved in oxidative stress tolerance. Here, we generated transgenic plants overexpressing *AtCYP21-4* and *OsCYP21-4* in potato and rice, respectively. The stems and roots of *AtCYP21-4–*overexpressing potato plants were longer than those of wild-type (WT) plants, which resulted in heavier tubers. *In vitro* tuberization in the transgenic potato also resulted in significantly greater tuber number and weight, as well as a shorter time to microtuber formation. Similarly, *OsCYP21-4–*overexpressing transgenic rice plants had higher biomass and productivity with longer early-stage internodes than the WT and higher seed weight. Immunoblot analysis with CYP21-4 antibody showed that these productivity-enhancing phenotypes were associated with high CYP21-4s protein expression. Anatomically, transgenic potato stems exhibited higher lignin content in xylem cells and thicker leaves. In addition, relative content of mannosidic glycoproteins per unit of total protein was above 20% in transgenic potato tubers and rice grains. Based on these findings, we propose that CYP21-4s are involved in the growth and development of plant vegetative and storage tissues via their effects on glycoprotein abundance or glycan processing in the Golgi apparatus. Thus, increasing CYP21-4s expression in crops could represent an alternative way to increase crop productivity and yield.

## Introduction

By 2050, the world's population will reach 9.7 billion according to a new UN report, and global grain demand is projected to double (Cassman, 1999; Tilman et al., 2002). The Green Revolution of the mid-twentieth century markedly increased agricultural productivity, largely via improvements in yield potential mediated by advances in disease, insect, and drought resistance (Ort et al., 2015). It is clear that global demand for food products will continue to increase, and that most of the increase in supply will need to be accomplished by increasing grain yield. These increases in agricultural productivity will mainly be accomplished through research aimed at improving genetic yield potential, development of agricultural technology, and minimization of loss and waste. Plant molecular biology and genetics will play important roles in satisfying this global demand. Currently, studies in these fields investigate the use of biotechnology to improve plant photosynthesis, water use efficiency, and biotic and abiotic stress resistance, with the goal of directly increasing yield or alleviating the impacts of water stress on biomass and yield.

Potato (*Solanum tuberosum* L.), which is grown around the world, is the fourth most important vegetable food crop, after corn, rice, and wheat, from the standpoint of stability of food supply and socioeconomic impact. Potato is vegetatively propagated through the “bud” or “eyes” present on tubers. Microtubers produced *in vitro* enable rapid multiplication of superior clones over a short period of time under controlled conditions. Consequently, microtubers are currently widely used as a source of material for disease-free germplasm exchange and higher-quality seed tubers (Donelly et al., 2003). This crop is ideal for the introduction of traits via biotechnology because it is amenable to propagation via tissue culture, and efficient transformation technology is available (Chakravarty et al., 2007). The recent full sequencing of the potato genome has significantly facilitated advances in potato biotechnology (Potato Genome Sequencing Consortium et al., 2011).

Rice (*Oryza sativa* L.), the most important staple food crop in Asia, has a much smaller genome than many other cereals, including maize and wheat. Consequently, rice is the main model crop for development of biotech plants (Kennedy, 2002) and accordingly, the genetic improvement of rice has attracted considerable attention from crop breeders, with the goal of increasing grain yield and quality. The rice genome has been sequenced (International Rice Genome Sequencing, 2005), and progress in plant science and biotechnology has led to major improvements in grain yield and agronomic traits.

The Golgi apparatus is a single membrane-bound organelle that is connected to a diverse array of other membrane-bound organelles, including the endoplasmic reticulum, plasma membrane, and endosomal compartments, via endomembrane systems mediated by vesicular and/or tubular transport carriers (Dacks et al., 2009). In plants, the Golgi apparatus plays essential roles in protein and lipid modification, sorting, and transport of macromolecules destined for secretory organelles and the extracellular milieu, and polysaccharide synthesis (Nebenfuhr and Staehelin, 2001; Dettmer et al., 2006; Park and Jurgens, 2011; Parsons et al., 2012b). Although, the practical modification steps may differ between species or cell types, the function of the Golgi apparatus as an assembly factory is conserved in all eukaryotes (Mellman and Simons, 1992). The essential biosynthetic functions of the Golgi apparatus include assembly and processing of *N*-linked and/or *O*-linked oligosaccharide side chains of glycoproteins, and in plant cells, this organelle is the site of *de novo* synthesis of complex cell wall matrix, hemicellulose and pectin polysaccharides (Driouich et al., 1993). Glycosylation and glycans play multiple biological roles in many physiological processes, including cell growth and differentiation, morphogenesis, and plant–pathogen and plant–environment interactions (Burn et al., 2002; Strasser et al., 2004, 2007; Kang et al., 2008; Von Schaewen et al., 2008; Chen et al., 2014). Thus, in-depth understanding and control of glycosylation of glycoproteins are becoming increasingly important in plant biotechnology applications.

Cyclophilins (CYPs) are intracellular receptors of the immunosuppressive drug cyclosporine A (Takahashi et al., 1989). They are categorized as members of the immunophilin family, which possess peptidyl prolyl *cis*/*trans* isomerase (PPIase) activity (Schmid, 1993). Proline is a unique amino acid that can adopt completely distinct *cis* and *trans* peptide bond conformations. Interconversion between the two conformations is controlled by PPIase enzyme activity (Lu et al., 2007), and the process of *cis*-*trans* isomerization plays a key role in the rate-limiting steps of protein folding (Wedemeyer et al., 2002). In plants, many immunophilins exhibit molecular chaperone function independent of PPIase activity (Kurek et al., 2002; Li and Luan, 2010). The representative model plants, Arabidopsis and rice have established classification systems for the immunophilin family of receptors (He et al., 2004; Ahn et al., 2010). Previously, we classified rice immunophilins, including 29 OsFKBPs and 26 OsCYPs (Ahn et al., 2010), and used reverse genetics to identify stress-responsive rice CYPs involved in salt, drought, photosynthetic, and cold stress (Park et al., 2013; Seok et al., 2014; Lee et al., 2015a,b, 2016; Yoon et al., 2016). In addition, rice CYP21-4 (OsCYP21-4) was identified as an unusual Golgi-localized immunophilin that contributes to oxidative stress tolerance by regulating peroxidase activity (Lee et al., 2015a). OsCYP21-4 was the first Golgi-targeted immunophilin to be functionally characterized in any organism.

In this study, we found that overexpression of *CYP21-4* orthologs (*AtCYP21-4* and *OsCYP21-4* in potato and rice, respectively) increased biomass and productivity during plant growth and development in both dicot and monocot crops. Anatomical characterization in CYP21-4-overexpressing transgenic plants revealed increased lignin content and leaf thickness. The glycoprotein assay revealed that, in the CYP21-4-overexpressing transgenic plants, ConA-enriched glycoprotein content was elevated in all tissues. Our results have important implications for the understanding of multifarious functions of the Golgi organelle in plant growth and development, and also highlight the importance of the CYP21-4 protein for crop improvement.

## Materials and methods

### Generation of AtCYP21-4–overexpressing transgenic lines

*In vitro* shoots of potato (*S. tuberosum* L. cv. Desiree) were maintained on culture plates (13.0 × 2.0 cm) containing basal MS (Murashige and Skoog, 1962) medium (pH 5.8) supplemented with 30 g/L sucrose and 8 g/L agar. Induction of *in vitro* shoots has been described previously (Park et al., 2004). *In vitro* cultures were maintained at 24 ± 1°C with 16 h photoperiod with a light intensity of 240 μmol photons m^−2^ s^−1^ provided by white fluorescent lamps. All procedures of potato transformation were carried out as previously reported (Kim et al., 2003). Briefly, inoculated leaf discs with *Agrobacterium* GV3101 harboring *AtCYP21-4* binary vector were placed onto basal MS medium containing 2.0 mg/L 2,4-D. Two days later, the explants were transferred to basal MS medium supplemented with 2.0 mg/L zeatin, 0.01 mg/L NAA, 0.1 mg/L GA3, 1,000 mg/L carbenicillin, and 100 mg/L kanamycin to allow shoot regeneration. Ten days after transfer, a callus formed at the wound region of the leaf fragments, and after 4 weeks, a small plant started to grow. The regenerated shoots that survived on antibiotic-containing selection medium were transferred to hormone-free medium for further rooting and molecular analysis. Genomic DNA, isolated from apical leaves of transgenic and WT plants, was analyzed by PCR with a forward primer complementary to *AtCYP21-4* and a reverse primer for the NOS-terminator, to confirm the presence of inserted 35S:*AtCYP21-4* (Table S1). The PCR conditions were as follows: 94°C for 3 min; 25 cycles of 94°C for 30 s, 55°C for 30 s, and 72°C for 30 s; and 72°C for 5 min.

### Measurement of transgenic potato plant growth and productivity

Whole tubers of similar size were planted in pots (1.5–2.5 cm diameter) during the growth period in a growth room (16 h light at 23°C/8 h dark at 19°C). Three-week-old plantlets from the growth room were transferred to 5,000 ml pots at 5 cm depth and grown in a greenhouse. Potato growth parameters, shoot length (cm) stem diameter (cm) and tuber number and weight (g) per plant after harvest were estimated. The tubers were grown for 90 days in ambient air. All parameters were measured in 10 plants per line, and three experiments were performed per parameter assayed.

### *In vitro* microtuberization of potato explant

Single-node cuttings or the plant itself dissected from 2-week-old axenic plantlets were cultured in basal MS medium (pH 5.8) supplemented with 90 g/L sucrose and 8 g/L agar in the dark at 20 ± 1°C to determine the tuberization time-course and tuber biomass, respectively (Kim et al., 1992). After 12 weeks, microtubers were harvested, and the number of microtubers per plant, as well as average and total weight (g) of microtubers, was recorded. *In vitro* microtuberization was conducted in 10 explants per line, and two biological experiments were performed.

### Growth and productivity measurement of transgenic rice plants

For growth and productivity measurements, *OsCYP21-4–*overexpressing transgenic plants, generated as described for the transgenic lines in a previous study (OE1–4; Lee et al., 2015a) were used. Seeds from transgenic lines and WT rice were sterilized with 70% ethanol and 50% chlorax, and then washed five times with sterilized deionized water. Seeds were sown on ½× MS media supplemented with 40 mg/L hygromycin B for selection of transgenic rice, and grown for 4 days in a growth chamber (28°C, 16 h light/8 h dark). Selected plants were transferred onto soil and grown for 2 weeks to investigate the growth phenotype of young plants. To survey reproductive-stage plants, 4-week-old rice plants were transferred to the field and grown from early June to late October. Seed weight was calculated by weighing 200 seeds per line. All parameters were measured in 10 plants per line, and three experiments were performed per parameter assayed.

### Semi-quantitative RT-PCR analyses of AtCYP21-4 transcription in transgenic potato plants

Total RNA was extracted from the leaves of *AtCYP21-4–*overexpressing transgenic and WT potatoes grown in a greenhouse using RNAiso Plus (TaKaRa, Tokyo, Japan). Total RNA treated with RNase-free DNase I (Fermentas, Burlington, Canada) was used for cDNA synthesis (RevertAid First-strand cDNA Synthesis Kit; Fermentas). Semi-quantitative RT-PCR was performed to determine *AtCYP21-4* transcript levels in the transgenic plants, as described by Yoon et al. (2016). Primers are listed in Table S1. The potato actin gene was used to normalize mRNAs among the samples (Table S1). PCR reactions were conducted under the following conditions: 95°C for 5 min; 30 cycles of 99°C for 30 s, 58°C for 30 s, and 72°C for 30 s; and extension for 5 min at 72°C.

### Histochemical staining

To examine the lignified cell walls in stems, AtCYP21-4–overexpressing transgenic and WT potato plants were grown in a growth room under 16 h light at 23°C/8 h dark at 19°C conditions for 2 months. The fourth internodes of stems from the ground level were excised and the internodes were sliced and subjected to histochemical analysis. For Wiesner staining, hand-cut stem sections were incubated in 1% phloroglucinol (w/v) in 6 mol/L HCl for 5 min, and then examined under a dissecting microscope (Pomar et al., 2002; Weng et al., 2010).

To identify anatomical traits that might be related to the growth physiology of AtCYP21-4–overexpressing leaves, small segments (5 × 5 mm) of lamina were excised from the same positions of all measured leaves (*n* = 3 for each line). The segments were fixed with 2.5% paraformaldehyde–glutaraldehyde buffered with 0.1 M phosphate (pH 7.2) for 2 h, postfixed in 1% osmium tetroxide in the same buffer for 1 h, dehydrated in graded ethanol and propylene oxide, and finally embedded in Epon-812. Ultra-thin sections, made with an ULTRACUT E (Leica, Wetzlar, Germany) ultramicrotome, were stained with uranyl acetate and lead citrate and examined under a CM 20 (Philips, Amsterdam, Netherlands) electron microscope.

### Total protein extraction and glycoprotein isolation

Total protein was extracted from leaves (vegetative tissue), tubers, and grains (storage tissues) of potato and rice plants using PBS buffer (137 mM NaCl, 2.7 mM KCl, 10 mM Na_2_HPO_4_, and 1.8 mM KH_2_PO_4_, [pH7.4]). Samples were ground in liquid nitrogen using a mortar and pestle, mixed with PBS buffer for 30 min on ice, and then centrifuged at 13,000 rpm at 4°C for 15 min. The supernatant was transferred to a new tube, and protein contents were quantified using Protein Assay Dye Reagent Concentrate (BIO-RAD, Hercules, CA, USA). Glycoprotein was isolated from total protein using the glycoprotein isolation kit–ConA (Thermo Fisher Scientific, San Jose, CA, USA). Before incubation, ConA resin was equilibrated with binding buffer, and 1 mg of total protein was incubated for 10 min at RT with the ConA resin. After the resin was washed four times, bound glycoproteins were eluted with elution buffer. Measurements of glycoprotein levels were performed in two biological replicates.

### Immunoblot assay

CYP21-4-specific peptides (Table S1) were used for immunize two rabbits. A company (Abfrontier, Seoul, Korea) carried out peptide synthesis, immunization, and antiserum purification. Immunoblot analysis was performed as described previously (Lee et al., 2015b). The prepared polyclonal CYP21-4 antibody was hybridized with total potato and rice proteins from the leaf tissue, and the CYP21-4 protein bands were confirmed as having the predicted size (determined from the mobility of recombinant OsCYP21-4 protein).

## Results

### Generation of *AtCYP21-4*–overexpressing transgenic potato plants

**Figure 1:**
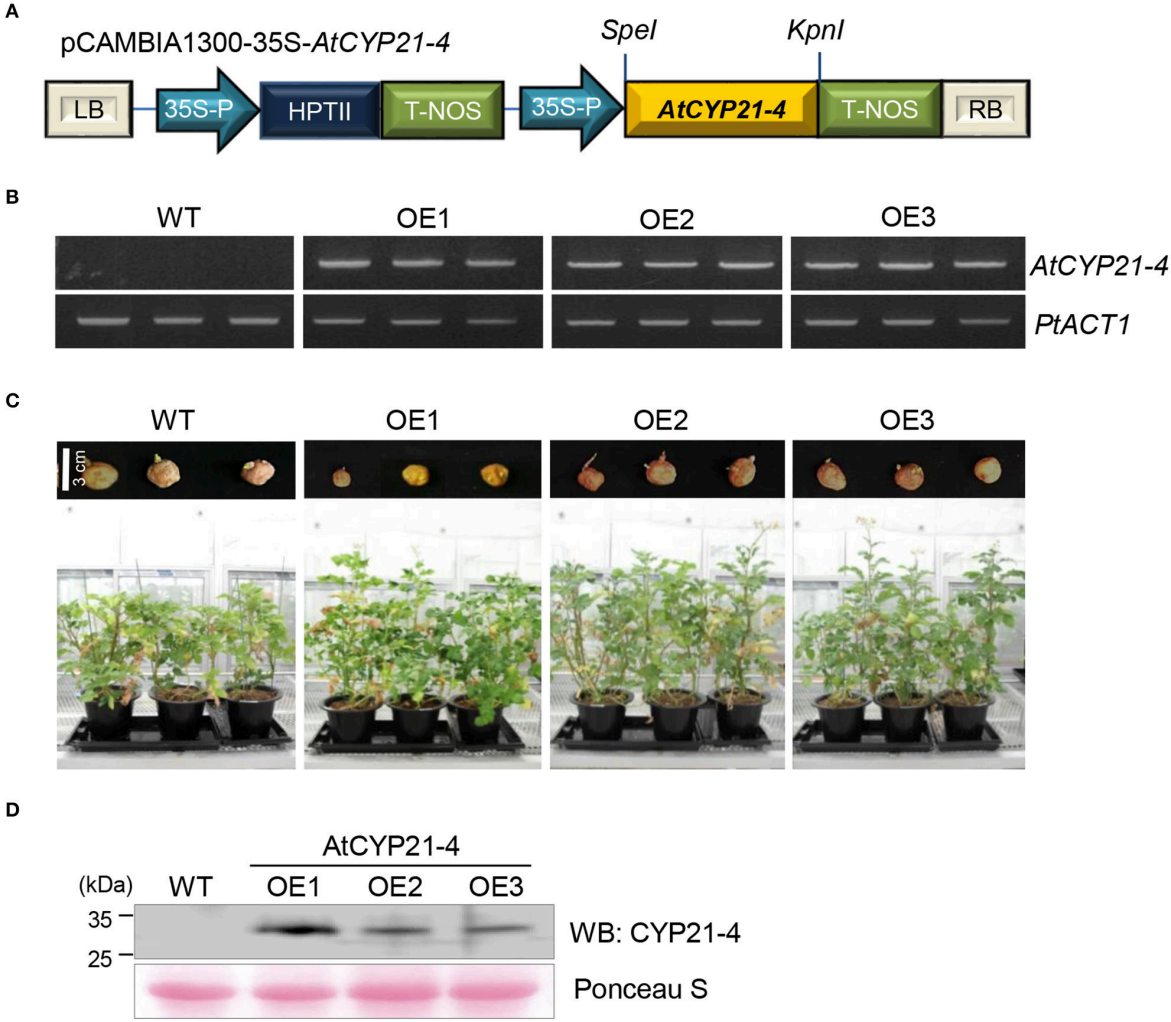
Expression of *AtCYP21-4* in transgenic potato plants. **(A)** Linear plasmid map of the pCAMBIA1300 binary vector harboring *AtCYP21-4* under the control of the CaMV35S promoter. **(B)** Ectopic expression of *AtCYP21-4* in potato. Semi-quantitative RT-PCR was performed on WT and *AtCYP21-4–*overexpressing transgenic potatoes. *PtACT1* was used as an internal control for mRNA normalization. **(C)** Effect on development of aerial parts in *AtCYP21-4–*overexpressing plants versus WT plants. **(D)** Immunoblot detection of AtCYP21-4 protein from independent *AtCYP21-4–*overexpressing lines. Total protein was extracted from the leaves of WT and *AtCYP21-4–*overexpressing potato plants. WT, wild-type potato cv. Desiree; OE1–OE3, independent AtCYP21-4–overexpressing transgenic plants.

To determine the effects of *CYP21-4* in potato, *AtCYP21-4* was used to generate transgenic plants. *AtCYP21-4* transformation vector was constructed as shown in Figure 1A. In this plasmid, *AtCYP21-4* gene expression was controlled by the CaMV 35S promoter. Using this construct, we generated three independent *AtCYP21-4–*overexpressing transgenic lines (OE1–OE3). To confirm *AtCYP21-4* overexpression in these three lines, we isolated total RNA from leaves and analyzed expression of *AtCYP21-4* by semi-quantitative RT-PCR. *AtCYP21-4* mRNA transcript was detected in all three transgenic lines, but not in wild-type (WT) potato (Figure 1B). To analyze the phenotypes of transgenic plants during growth development, whole tubers of similar size were planted into pots, and each line was grown in greenhouse conditions. During the growth process, *AtCYP21-4–*overexpressing potatoes exhibited significantly enhanced development of aerial parts in comparison with WT potato, 3 months after the tubers were planted (Figure 1C). To ascertain whether the growth phenotype of the transgenic plants was a consequence of the elevated CYP21-4 protein level, we performed immunoblot analysis using an antibody against CYP21-4, developed in our laboratory. Figure 1D clearly shows expression of AtCYP21-4 protein in all potato transgenic lines; however, the level of CYP21-4 protein expression and the phenotype of the transgenic plant did not correlate in a straightforward manner.

### AtCYP21-4–overexpressing transgenic plants exhibit elevated biomass and productivity

**Figure 2:**
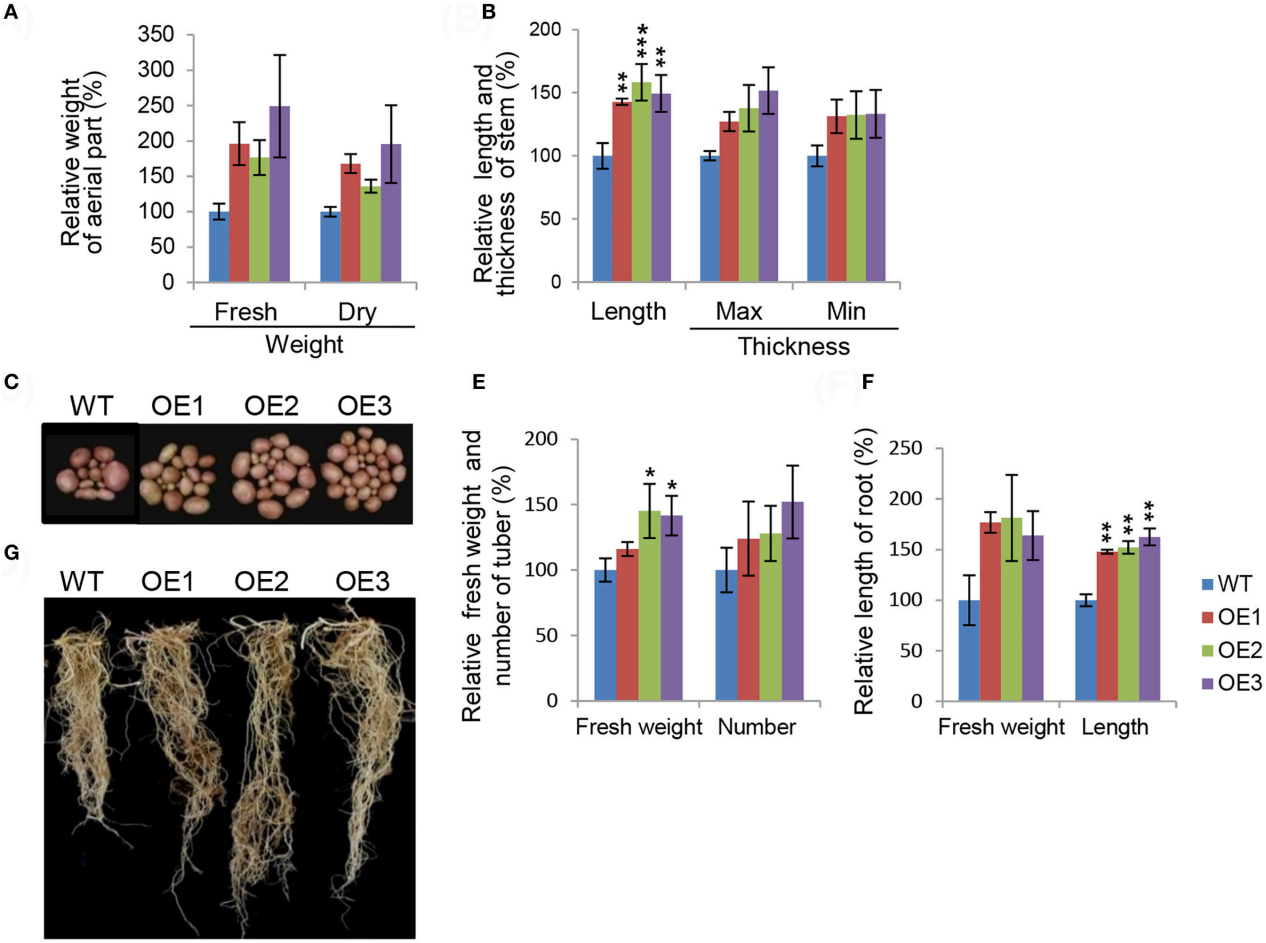
Morphological characteristics of AtCYP21-4–overexpressing transgenic potato plants. Comparison of growth in aerial parts **(A,B)**, and tuber and root **(C–F)** of the WT and the AtCYP21-4–overexpressing transgenic lines (OE1, OE2, OE3). Both fresh and dry weight of aerial parts were elevated in the transgenic potatoes, and stem length and thickness were greater than in the WT **(A,B)**. Transgenic potatoes produced larger numbers of tubers and higher fresh tubers weights than the WT plants **(C,E)**. Transgenic potatoes had longer roots and higher fresh weights than the WT **(D,F)**. WT, wild-type potato cv. Desiree; OE1–OE3, independent AtCYP21-4–overexpressing transgenic plants. Data were analyzed using ANOVA followed by Dunnett's test. Error bars represent SE of three biological replicates (^*^p < 0.05; ^**^p < 0.01 *n* = 10).

To characterize phenotypes in a more concrete manner, we assessed major growth traits during development stages in the transgenic potatoes. Overall biomass production was remarkably elevated in transgenic potatoes (Figure 2). The difference in fresh weight was not statistically significant because there was considerable inter-individual variation in fresh weights (Figure 2A). The maximum and minimum diameters of the main stems were also not significantly different from those of WT plants, while stem length was up to 1.5-fold longer than in WT plants (Figure 2B). These observations support the idea that AtCYP21-4 affects growth of the aerial part of the plant. At harvest time, we evaluated agricultural traits in the subterranean parts of the transgenic potatoes (Figures 2C–F). For this purpose, tuber and root images of transgenic plants OE1–OE3 and the WT were acquired (Figures 2C,D). In comparison with the WT, transgenic lines exhibited increases in tuber productivity, including total fresh weight, with OE3 having the highest values (Figure 2E). To determine whether the vigorous growth and tuber productivity of the overexpression lines was affected by the root tissue growth, we measured root biomass in the transgenic plants. Root were much longer in the transgenic lines than in the WT, as shown in Figure 2F. Thus, AtCYP21-4 protein plays a role in plant growth and crop productivity in potato.

### Overexpression of AtCYP21-4 promotes formation of *In vitro* tuberization

**Figure 3:**
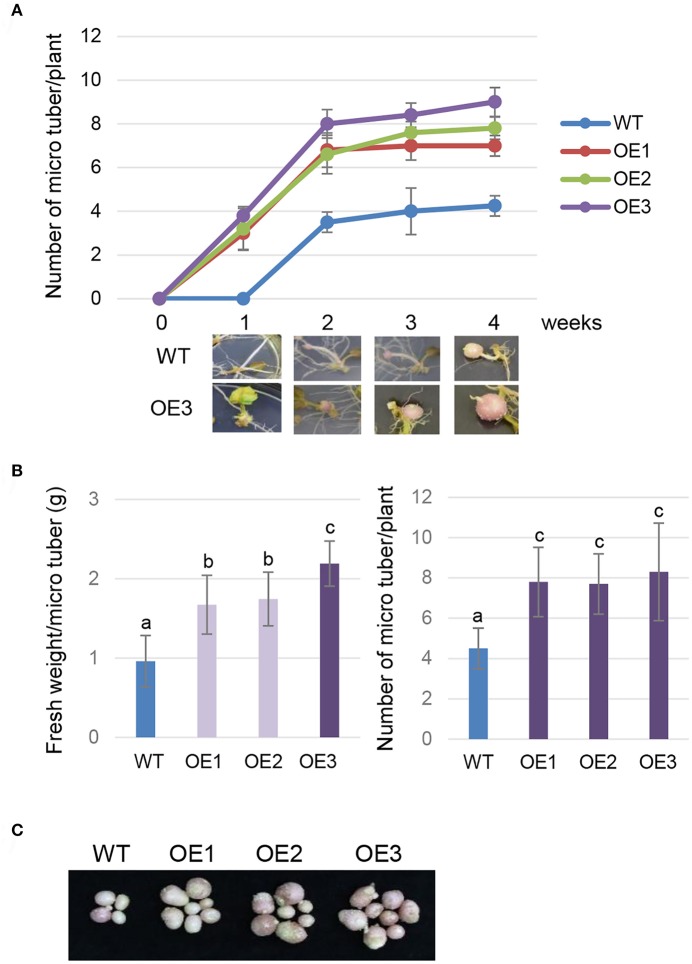
*In vitro* tuberization of transgenic plants. Time-course tuberization rate of the wild-type (WT) and the AtCYP21-4 transgenic lines (OE1, OE2, OE3) **(A)**. Number of microtubers was determined at the indicated culture times under tuber-inducing conditions (upper panel), and images of microtubers were photographed at the same times (lower panel). AtCYP21-4–overexpressing plants exhibited faster tuber initiation rates than the WT, and most transgenic plants developed significantly shorter stolons than WT ones. Fresh weight (g) per microtuber and number of microtubers per plant **(B)**, and photocopies of harvested microtubers **(C)** were investigated at harvest 12 weeks after *in vitro* culture. Transgenic potatoes generated more microtubers, and had a higher fresh weight than the WT plants. Data were analyzed by means of analysis of variance (ANOVA), and the means were compared by the Duncan's multiple range test (DMRT) at *P* = 0.05.

To evaluate the effect of AtCYP21-4 overexpression on tuberization and tuber biomass, we cultured single-node cuttings or the plant itself from the three transgenic lines and the WT in tuberization medium under darkness. The tuberization process was assessed every week, and the fresh weight of mature tubers was measured after 12 weeks. Time-course *in vitro* tuberization analysis using single-node cuttings revealed that AtCYP21-4 OE lines had faster tuber initiation rates than the WT 1 week after the start of the experiment, with OE3 forming tubers earliest (Figure 3A). Microtubers developed from the transgenic lines *in vitro* were extremely different from those of the WT. The WT microtubers had long stolons far from the axillary bud, whereas transgenic explants of all three lines formed tubers attached directly to the axillary bud, with no stolon growth. The promotion of tuberization resulted in higher fresh weight per tuber (Figure 3B, left). Furthermore, the transgenic explants had roughly twice as much mean numbers of tubers per plant than the WT (Figure 3B, right). Figure 3C shows that number and size of microtubers harvested from the overexpression lines were greater than those of microtubers harvested from the WT. This result indicates that AtCYP21-4 stimulates tuber induction, and increases the biomass of potato tubers. The underlying mechanism will be investigated in more detail in a future study.

### *OsCYP21-4*–overexpressing transgenic rice exhibited elevated internode elongation at very early stages

**Figure 4:**
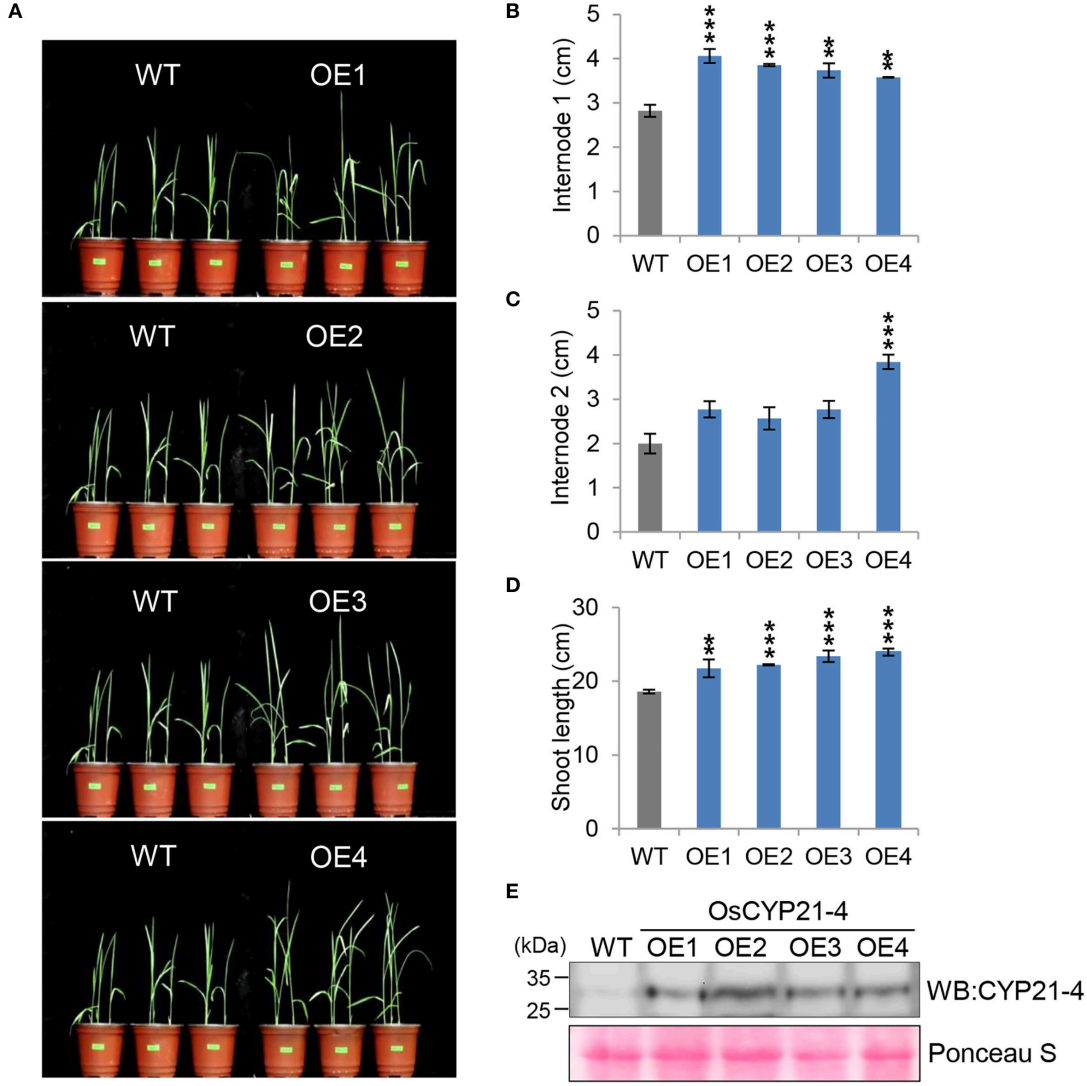
Growth phenotypes of *OsCYP21-4–*overexpressing transgenic rice in young plants. **(A)** WT and *OsCYP21-4* OE transgenic rice were grown in a growth chamber at 28°C under 16 h light/8 h dark conditions for 2 weeks. **(B–D)** At the same time, internode 1 **(B)**, 2 **(C)**, and shoot lengths **(D)** of WT and *OsCYP21-4* OE transgenic rice were measured from 10 plants per line. Error bars indicate SE of three biological replicates. Data were analyzed using ANOVA followed by Dunnett's test. Error bars represent SE of three biological replicates (^**^p < 0.01; ^***^p < 0.001, *n* = 10). **(E)** Total protein extracted from leaf tissue of WT and *OsCYP21-4–*overexpressing plants was subjected to immunoblot assay using an anti-CYP21-4 antibody. WT, wild-type rice cv. Dongjin; OE1–OE4, OsCYP21-4–overexpressing transgenic rice (OE1–OE4).

In a recent study, we generated transgenic rice overexpressing the *OsCYP21-4* gene under the control of the CaMV 35S promoter, and showed that *OsCYP21-4–*overexpressing transgenic rice is tolerant to oxidative stress (Lee et al., 2015a). To determine the function of *CYP21-4* in plant growth and development in a monocotyledonous crop, we investigated the growth phenotype of four independent *OsCYP21-4–*overexpressing lines (OE1–OE4) under normal growth conditions. Because the overexpression lines exhibited a marked increase in initial growth (Figure 4A), we calculated the lengths of shoots and internodes of the WT and OE1–4 transgenic plants. At the 2-week-old stage, the average first internode length (internode 1) in the WT was about 2.8 cm, whereas those in the transgenic lines were 3.5–4 cm (Figure 4B). The second internode length (internode 2) of *OsCYP21-4–*overexpressing lines was not statistically significant (Figure 4C). As a result, the shoot lengths of transgenic plants were up to 1.3-fold longer than those of the WT plants at this stage (Figure 4D). In addition, we measured OsCYP21-4 protein levels in the transgenic plants by immunoblotting with a CYP21-4 antibody. OsCYP21-4 proteins were unambiguously more abundant in OE1–4 than in WT plants, and expression was similar among all four transgenic lines (Figure 4E). These data suggest that OsCYP21-4 is also involved in early growth development in rice.

### OsCYP21-4–overexpressing transgenic rice also show increases in biomass and productivity

**Figure 5:**
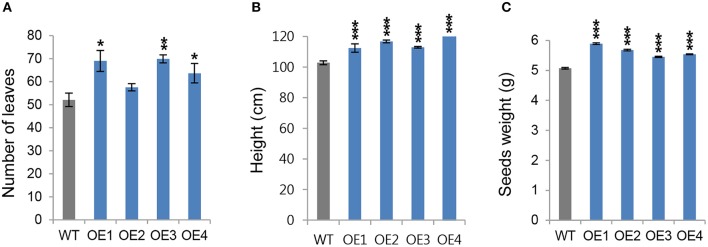
Productivity of OsCYP21-4 overexpressing transgenic rice at reproductive stages. **(A–C)** WT and OsCYP21-4–overexpressing rice (OE1–OE4) were grown in the field at 28°C for 14 weeks. The number of leaves **(A)**, and height **(B)**, were measured from 10 plants per line. **(C)** After seed harvest, seed weight was calculated from 200 seeds per line. Seed weights were measured from 10 plants per line. WT, wild-type rice cv. Dongjin; OE1–OE4, OsCYP21-4–overexpressing transgenic rice (OE1–OE4). Data were analyzed using ANOVA followed by Dunnett's test. Error bars represent SE of three biological replicates (^*^p < 0.05; ^**^p < 0.01; ^***^p < 0.001, *n* = 10).

Next, we wanted to determine whether the growth phenotype observed in young OsCYP21-4 transgenic plants was retained until the reproductive stage. To obtain additional information about the function of OsCYP21-4 in mature plants, we grew transgenic and WT plants in the field for 14 weeks, and monitored the number of leaves as well as height of shoot. When the plants were harvested at 32 weeks after planting, seed weight was measured to determine productivity. The number of leaves was higher in most of the OsCYP21-4 overexpressing transgenic lines, whereas the OE2 line did not differ significantly from the WT. On average, transgenic plants had greater numbers of leaves (20%) than the WT (Figure 5A). Also, all of the overexpression lines were significantly taller (10–20 cm) than the WT plants (Figure 5B). In the OsCYP21-4–overexpressing lines, seed weight, a trait that is critical for the productivity of rice plants, was greatly increased (10–15% higher than in the WT; Figure 5C). These data suggest that OsCYP21-4 also increases rice plant biomass and productivity by facilitating plant growth.

### AtCYP21-4–overexpressing potato exhibits elevated lignin composition and leaf thickness

**Figure 6:**
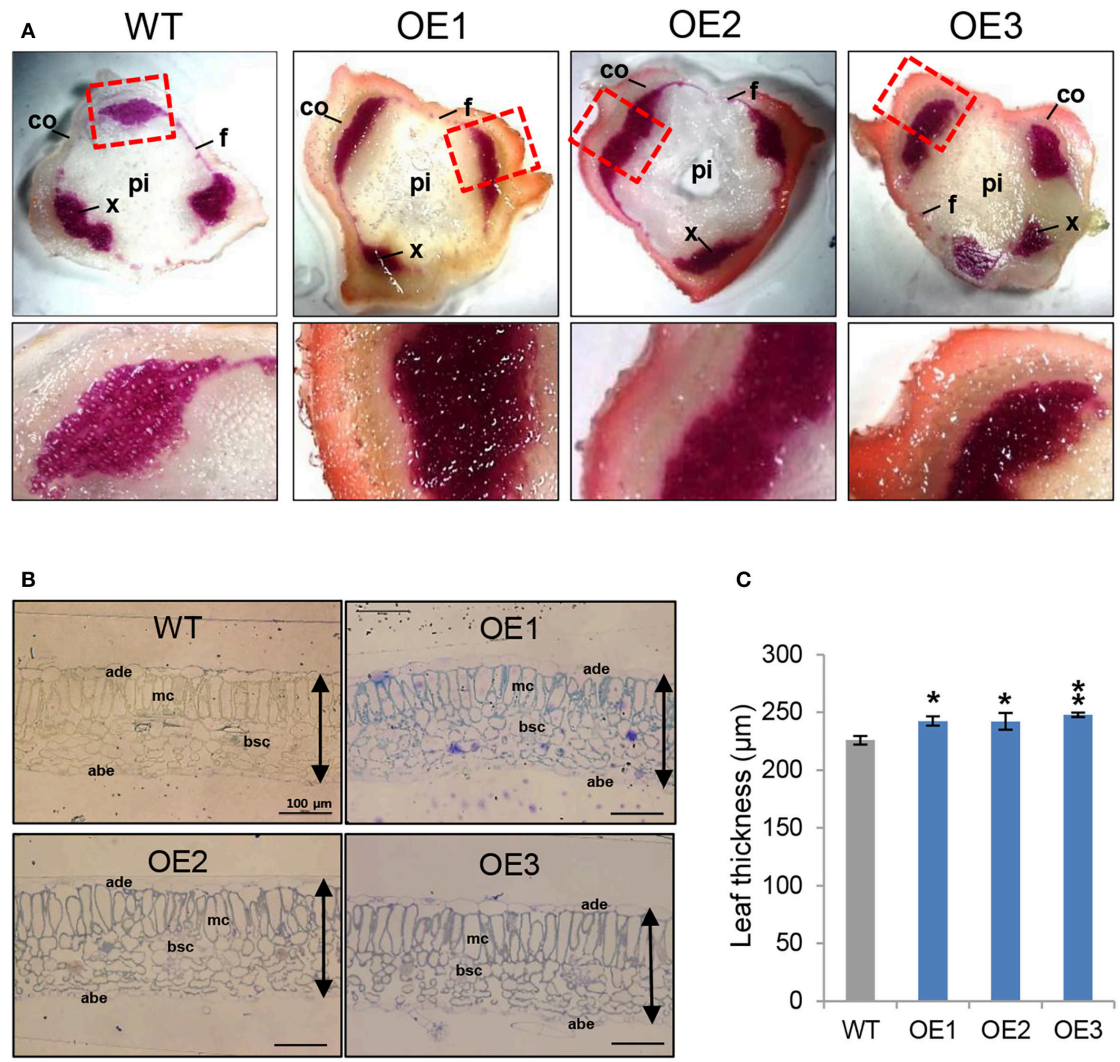
Stem lignin composition and leaf thickness of AtCYP21-4–overexpressing potato plants. **(A)** Transverse sections of stems were stained with phloroglucinol-HCl to detect lignin. The phloroglucinol-HCl reagent detects aldehyde groups contained in lignin and yields a red staining that is generally indicative of the presence of lignin. Lignin staining was heavy in both xylem cells and interfascicular fibers. Co, Cortex; f, interfascicular fiber; pi, pith; x, xylem. Magnification × 20. **(B)** Cross-sections of leaf tissues from WT and AtCYP21-4 transgenic potato plants. Bars represent 100 μm in all cases. WT, wild-type potato cv. Desiree; OE1–OE3, independent AtCYP21-4–overexpressing transgenic plants. **(C)** Leaf thickness was calculated by measuring the length from adaxial epidermis to abaxial epidermis. ade, adaxial epidermis; abe, abaxial epidermis; mc, methophyll cells; bsc, bundle sheath cells. Bars indicate standard deviation of three replicates. Data were analyzed using ANOVA followed by Dunnett's test (^*^p < 0.05; ^**^p < 0.01, *n* = 3).

AtCYP21-4–overexpressing potatoes had generally higher levels of biomass in both the aerial and subterranean parts of the plant. Because a prominent feature of the aerial parts of the transgenic plants was an increase in stem thickness, we examined the possible anatomical impacts of *AtCYP21-4* gene on stem growth and development. To determine whether biomass and productivity of the transgenic plants were increased by the effect of CYP21-4 on secondary growth, which is marked by an increase in plant thickness, we investigated the lignin content of the stem tissues. Lignin, an aromatic biopolymer, is a component of the secondary xylem, which provides hardiness and strength (Boerjan et al., 2003). We prepared thin sections from the stems of the fourth nodes in the main stems of 2-month-old plants and stained them with phloroglucinol-HCl, which yields a violet-red color indicative of total lignin. Xylem cells and interfascicular fibers in the stems stained positive for lignin, showing that it had been specifically deposited in these areas, whereas cortical cells or pith parenchyma cells did not. The violet-red color was stronger in the transgenic stems than in the WT (Figure 6A). In addition, Mäule stain, which is specific for syringyl lignin units in xylem and interfascicular fibers, also revealed more dark brown staining in transgenic than the WT stems (Figure S1). The results of the staining experiments clearly demonstrated that lignin content was higher in the overexpression potato lines than in the WT.

To determine whether anatomical changes also occurred in the leaf tissue, we conducted microscopic analysis of leaf thickness in WT and overexpression lines. Specifically, we measured leaf thickness from the adaxial epidermis to the abaxial epidermis. We detected a statistically significant increase of about 10% in transgenic vs. WT leaves; however, no morphological differences were apparent between transgenic and WT plants (Figures 6B,C). Microscopic observations of the epidermal tissues of transgenic potato leaves showed that overexpression of CYP21-4 did not affect cell division (data not shown); thus, we believe that the increase in leaf thickness was due to a slight increase in cell size.

Together, the observations reported in this section demonstrate that AtCYP21-4 overexpression increases stem and leaf thickness, implying that CYP21-4 is involved in secondary growth.

### *CYP21-4* is involved in glycoprotein regulation in the golgi apparatus

In a previous study, we reported that OsCYP21-4 protein is targeted to the Golgi apparatus. Here, we confirmed the subcellular localization of AtCYP21-4, the *Arabidopsis* homolog, using GFP-fused AtCYP21-4 protein introduced by agro-infiltration into *Nicotina benthamiana* protoplasts. As predicted, AtCYP21-4 protein was also localized to the Golgi apparatus, as ascertained by localization with mannosidase-mCherry Golgi marker protein (α-ManI-mCherry; Figure S2). Almost all glycosylation reactions occur in the Golgi apparatus, and multiple glycosyltransferases and glycosidases reside there (Stanley, 2011). Accordingly, we sought to determine whether CYP21-4 is involved in glycoprotein regulation.

**Figure 7:**
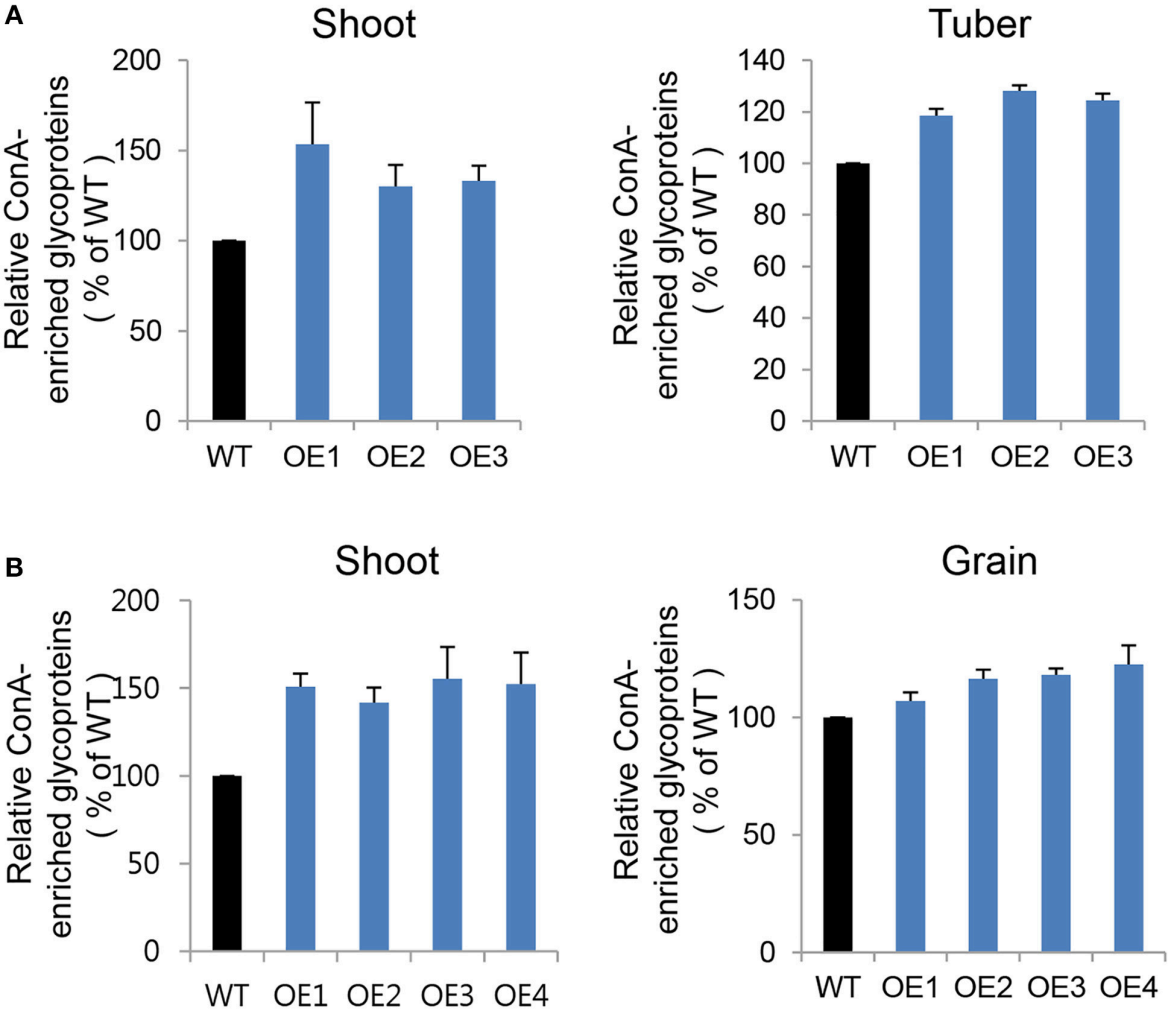
Glycoproteins in endogenous ConA-purified proteins from transgenic potato plants. **(A)** Relative ConA-enriched glycoprotein contents of shoot and tuber tissues from WT and AtCYP21-4–over expressing potato plants. WT, potato wild type cv. Desiree; OE1–OE3, independent AtCYP21-4–overexpressing transgenic potatoes. **(B)** Relative ConA-enriched glycoprotein contents of shoot and grain tissues from WT and OsCYP21-4–over expressing rice plants. WT, rice wild-type cv. Dongjin; OE1–OE4, independent OsCYP21-4–overexpressing transgenic rice plants. For each sample, 1 mg of total protein was purified using ConA resin. Error bars denote SE of two biological replicates.

To compare glycoprotein content between transgenic and WT plants in both potato and rice crops, we concentrated glycoproteins from equal amounts of protein extract using ConcanavalinA (Con A), a lectin-bearing resin that preferentially binds to α-mannose and α-glucose-containing proteins (Mustafa and Komatsu, 2014). In our comparative analysis of glycoprotein contents, we investigated both vegetative (shoot) and storage tissues, including tuber (potato) and grain (rice). Mannosidic glycoprotein contents were significantly higher in CYP21-4 OE potato shoot (30%) and tuber (25%), as well as in OE rice shoot (45%) and grain (11%), than in the WT plants (Figures 7A,B). Each transgenic line exhibited ConA-enriched glycoprotein content differences that varied depending on the vegetative or storage tissue. The major potato tuber protein was patatin, which belongs to a family of 40–42 kDa glycoproteins that represent around 40% of total soluble protein in tubers. ConA-column chromatography revealed that patatin was present in higher amounts in AtCYP21-4–overexpressing potato tubers (Figure S3). These results suggest that CYP21-4 plays a tissue-specific role in the regulation of mannosidic glycoproteins or in their glycan processing in the Golgi.

## Discussion

Even though, a previous study functionally linked *OsCYP21-4* to oxidative stress tolerance (Lee et al., 2015a), a great deal remains unknown about the biological and physiological roles of CYP21-4 in plant growth and development. In this study, we revealed that CYP21-4s play functional roles in plant development in both monocot and dicot crops. In potato, a dicot, *AtCYP21-4* was used for functional analysis of CYP21-4 (Figures 1A,B), and the Golgi organelle localization of the protein in plant cells was confirmed (Figure S2). All transgenic plants in which AtCYP21-4 was ectopically expressed exhibited elevated growth in both aerial and underground parts, in comparison with the WT, resulting in higher crop productivity (Figures 1C, 2A–G). OsCYP21-4–overexpressing rice lines also exhibited similar elevated growth in both vegetative (Figure 4) and reproductive (Figure 5) developmental stages. In particular, the early stages of internode growth in rice were significantly affected by overexpression of OsCYP21-4 (Figures 4B,C). Together, these results uncover an important role for CYP21-4 proteins in major crops and inter-class correlation between monocot and dicot. Immunoblots revealed that CYP21-4 proteins were produced at high levels in the transgenic plants, and suggested that CYP21-4 plays an important role in plant growth and development (Figures 1D, 4E). Despite the paucity of studies on the growth and developmental functions of Golgi-targeted proteins in plants, with the exception of the glycan biosynthesis proteins, the recent demonstration that GDP-fructose transporter 1 in Arabidopsis is indispensable for plant growth and development (Rautengarten et al., 2016), along with the results of this study, suggests that Golgi-localized proteins play diverse roles in plant growth and development. The data presented here and in previous studies provide evidence for the hypothesis that CYP21-4, a Golgi protein, contributes to growth and development associated with oxidative stress in terrestrial plant species.

Microtubers are an advantageous source of disease-free material for storage and transportation (Zheng et al., 2012). A great deal of research effort has been expanded to determine the best conditions for *in vitro* microtuber production; this work revealed that microtuberization is controlled by several factors such as photoperiod, sucrose, hormones and temperature (Vreugdenhil and Struik, 1989; Coleman et al., 2001; Dieme and Sy, 2013; Kumlay, 2014; Al-Hussaini et al., 2015). We performed *in vitro* propagation of microtubers to investigate whether CYP21-4 overexpression has a positive effect on the microtuberization process. Interestingly, AtCYP21-4–overexpressing explants produced more microtubers (Figures 3B,C), and the period of microtuber formation was shorter than in the WT (Figure 3A). Previous work identified sucrose transport and starch biosynthesis–related genes that induce tuber initiation and growth (Navarre and Pavek, 2014). Therefore, given that starch accumulation is an important component of tuberization, we cannot exclude the possibility that CYP21-4 plays a role in a mechanism such as sucrose transport or regulation of sucrose-to-starch conversion. Further molecular and physiological studies of the function of CYP21-4 in the Golgi during microtuber development will improve our understanding of the regulatory mechanisms impacting starch accumulation in potato tuberization.

We found elevated deposition of lignin in stems and a slight increase in leaf thickness in AtCYP21-4–overexpressing transgenic plants (Figures 6, Figure S1), indicating that the *CYP21-4* gene is involved in secondary growth. However, a further cytological study will be necessary to identify the role of CYP21-4 in increasing productivity. Furthermore, CYP21-4–overexpressing transgenic plants showed increases in mannosidic glycoprotein content throughout vegetative and storage tissues, in both potatoes and rice (Figure 7). Although it is not yet known whether CYP21-4 plays a causal role in glycosylation maturation within the Golgi, our preliminary analysis of *N*-glycan complexes in potatoes using ConA binding and the HRP antibody (data not shown) suggests that CYP21-4 influences protein glycosylation in the Golgi apparatus. Protein glycosylation, the most prevalent post-translational modification of secretory and membrane proteins in the Golgi, can directly affect folding, sorting, stability, and interactions of proteins, and thus plays a crucial role during plant growth and development, as well as in the response to various stress conditions. In plants, mutants with glycan deficiencies exhibit embryonic lethality, altered immunity, shoot and root growth defects, and stress-sensitive phenotypes (Zhang et al., 2009; Farid et al., 2011; Qin et al., 2013; Dai et al., 2014; Wang et al., 2014; Blanco-Herrera et al., 2015). A growing body of evidence strongly supports the distinct roles of glycosylation in plant growth and development, although little is known regarding the underlying mechanisms. Further studies are necessary to investigate in detail the biological functions of CYP21-4 in the Golgi apparatus, including its roles in glycosylation processes, throughout plant growth and development.

In studies of plant immunophilins, CYP21-4s were classified as mitochondria-localized proteins, based on the results of prediction software (He et al., 2004; Ahn et al., 2010). However, present (AtCYP21-4) and previous (OsCYP21-4) studies confirmed that CYP21-4 is localized to the Golgi apparatus (Figure S2). More recently, Golgi proteome analysis based on large-scale quantitative and cytological enrichment has been employed to assess the relative abundances of Golgi-localized proteins, and ultimately to enable studies centered on the biochemical and cell biology aspects of the Golgi (Nikolovski et al., 2012; Parsons et al., 2012a). A number of CYPs, including CYP21-4, were detected by isotope tagging (LOPIT) and free-flow electrophoresis (FEE; Nikolovski et al., 2012; Parsons et al., 2012a). These results suggest that post-translational regulation of CYPs may be involved in various essential roles of the Golgi apparatus. Once a given CYP is confirmed to localize to the Golgi, it will be interesting to determine whether it functions as a PPIase, foldase, chaperone, or signaling molecule.

## Author contributions

HC and HK conceived and designed the study and wrote the manuscript. HP, AL, and KM performed the experiments and wrote the manuscript. DA and SL conducted potato transformation and analysis. JA contributed to scientific discussions and revision of the manuscript.

### Conflict of interest statement

The authors declare that the research was conducted in the absence of any commercial or financial relationships that could be construed as a potential conflict of interest.
