# Supplementary material for: Overexpression of Golgi Protein CYP21-4s Improves Crop Productivity in Potato and Rice by Increasing the Abundance of Mannosidic Glycoproteins
Source: Front Plant Sci. 2017 Jul 20;8:1250. doi: 10.3389/fpls.2017.01250 (PMC5517489; doi:10.3389/fpls.2017.01250)
Supplement: Supplementary file 1 [file Table1.docx]

**Table S1. Primer and peptide sequences used in this study**

| **Name** | **Primer sequences**  **(F, forward; R, reverse [5'-3'] )** | **Applications** |
| --- | --- | --- |
| *AtCYP21-4*  (pCAMBIA1300) | F :CCGGGATCCATGGCGAAGATAAAACCGCAGGCT | Transgenic plant, RT-PCR, |
|  | R :AACGTCGACTCATGTCTCTAGTTTCAGTACAAC |  |
| *PtACT1* | F : TCAGTAGGACCACATGACCCTG | RT-PCR, |
|  | R : GAACAATCCAGGCTCGCATTTG |  |
| *OsCYP19-4*  (pCAMBIA1300) | F : GAATTCATGGCGAGGATAAAGCCGAAGCAATTG | Transgenic plant |
|  | R :GGATCCTCAGCTCAAAGCTTGCTGTTTTAGCGTGATG |  |
| *AtCYP21-4*  (pCAMBIA1302) | F :CCGCCATGGCCATGGCGAAGATAAAACCGCAGGCT | Subcellular localization |
|  | R :AACACTAGTTGTCTCTAGTTTCAGTACAACCTCTG |  |
| Polyclonal  anti-CYP19-4 antibody | Peptide 1 : DLPRAEHVGRSEDSTKTSRPSY | Immunoblot |
|  | Peptide 2 : VQEIEEVDTDEHYQPKTPIG |  |
