# Supplementary material for: Overexpression of Golgi Protein CYP21-4s Improves Crop Productivity in Potato and Rice by Increasing the Abundance of Mannosidic Glycoproteins
Source: Front Plant Sci. 2017 Jul 20;8:1250. doi: 10.3389/fpls.2017.01250 (PMC5517489; doi:10.3389/fpls.2017.01250)
Supplement: Supplementary file 2 [file Presentation1.pdf]

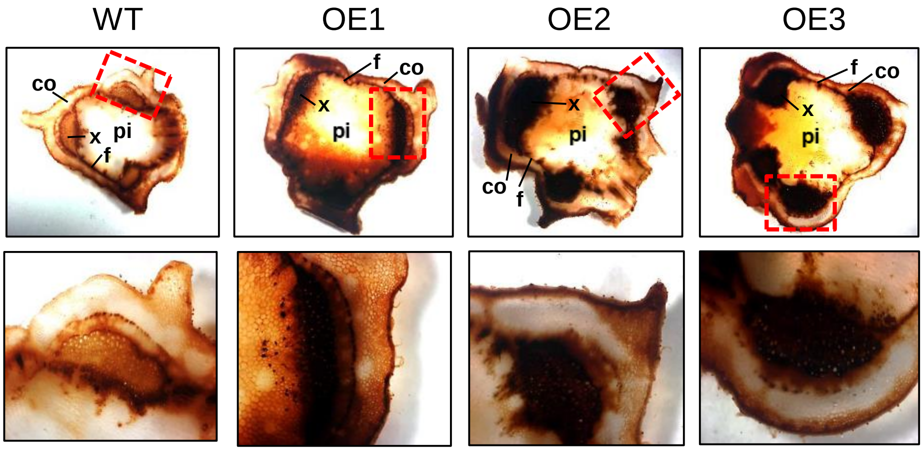

Figure S1. Mäule staining of Wild-type and AtCYP21-4 overexpressing potato stem cross-sections. Transverse sections of stems were stained with Mäule staining for the detection of lignin. Mäule staining (dark brown color) of wild-type and transgenic stems section showing the normal lignin deposition in the walls of xylem cells and interfascicular fibers. Co, Cortex; f, interfascicular fiber; pi, pith; x, xylem. Magnification, ×20. WT; wild-type potato, OE1~OE3; independent AtCYP21-4 overexpressing transgenic potato.

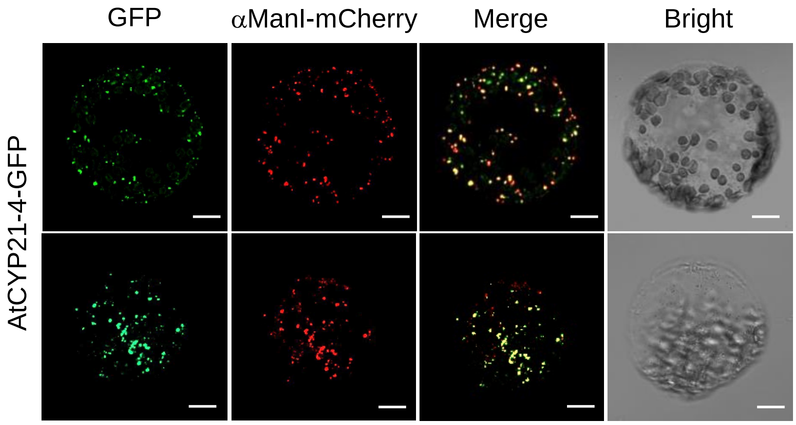

Figure S2. The subcellular localization of AtCYP21-4 protein in the *Nicotiana benthamiana* protoplasts. Agrobacterium (GV3101) transformed AtCYP21-4-GFP construct infiltrated into the leaf of 5-week-old *Nicothana benthamiana*. Two days after infiltration, protoplasts were isolated by enzymatic treatment (1% cellulose and 0.25% macerozyme) of leaves for 6 hr and fluorescence imaging was carried out using a Zeiss R510 confocal laser microscope. α-Mannosidase-mCherry is used for Golgi-resident marker protein. Bars = 10 μm.

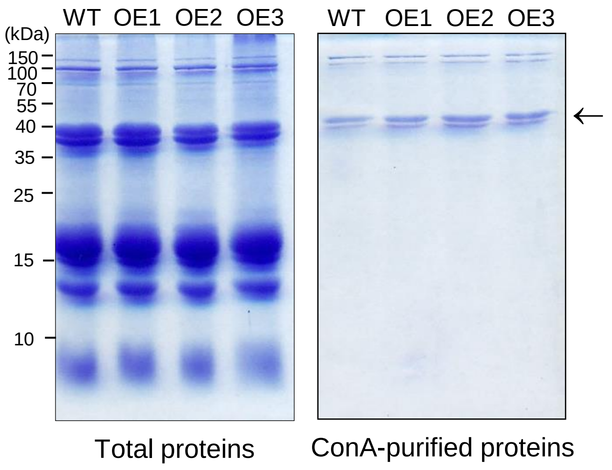

Figure S3. Protein extraction and ConA-enriched glycoprotein purification from transgenic potato tubers. Total soluble proteins were extracted from the tubers of wild type and AtCYP21-4 overexpressing potato plants using PBS buffer and 30 μg of total proteins were loaded per lane. 12% SDS-PAGE gel was stained with Coomassie staining solution (left). 1 mg of total soluble proteins were purified using a ConA-purification resin and eluted equal volume. 10 μl of ConA purified elutes were loaded on gel and stained (right). An arrowhead indicates estimated patatin protein bands (about 40 kDa). WT; wild-type potato, OE1~OE3; independent AtCYP21-4 overexpressing transgenic potato.
